# Involvement of *Atm* and *Trp53* in neural cell loss due to *Terf2* inactivation during mouse brain development

**DOI:** 10.1007/s00418-017-1591-3

**Published:** 2017-06-15

**Authors:** Jusik Kim, Inseo Choi, Youngsoo Lee

**Affiliations:** 1 Genomic Instability Research Center, School of Medicine, Ajou University, Suwon, 16499 Republic of Korea; 2 Department of Biomedical Sciences, The Graduate School, Ajou University, Suwon, 16499 Republic of Korea

**Keywords:** Atm, DNA damage, Apoptosis, Brain development

## Abstract

Maintenance of genomic integrity is one of the critical features for proper neurodevelopment and inhibition of neurological diseases. The signals from both ATM and ATR to TP53 are well-known mechanisms to remove neural cells with DNA damage during neurogenesis. Here we examined the involvement of *Atm* and *Atr* in genomic instability due to *Terf2* inactivation during mouse brain development. Selective inactivation of *Terf2* in neural progenitors induced apoptosis, resulting in a complete loss of the brain structure. This neural loss was rescued partially in both *Atm* and *Trp53* deficiency, but not in an *Atr*-deficient background in the mouse. *Atm* inactivation resulted in incomplete brain structures, whereas *p53* deficiency led to the formation of multinucleated giant neural cells and the disruption of the brain structure. These giant neural cells disappeared in *Lig4* deficiency. These data demonstrate ATM and TP53 are important for the maintenance of telomere homeostasis and the surveillance of telomere dysfunction during neurogenesis.

**Electronic supplementary material:**

The online version of this article (doi:10.1007/s00418-017-1591-3) contains supplementary material, which is available to authorized users.

## Introduction

Genomic instability resulting from DNA damage induced by either endogenous or exogenous insults could lead to defective neurodevelopment and neurological diseases (Lee et al. 2016). Ataxia telangiectasia mutated (ATM) is one of the early responders to DNA damage, particularly to DNA double-strand breaks (DSBs). ATM mutations cause Ataxia Telangiectasia (A-T) characterized by ataxia due to loss of Purkinje and granule cells in the cerebellum (McKinnon 2012, 2013). Similarly, Ataxia-telangiectasia and RAD3-related (ATR) recognizes single-stranded DNA resulting from replication stress. Seckel syndrome 1 (SCKL1) due to hypomorphic mutations in the *ATR* gene is characterized by microcephaly and mental retardation (Nam and Cortez 2011; McKinnon 2013). Once activated by DNA damage, ATM and ATR phosphorylate several overlapping substrates including tumor protein p53 (TP53/Trp53 in mice) to regulate DNA damage repair, apoptosis, and cell cycle arrest (Lee et al. 2001, 2012b; Lovejoy and Cortez 2009). Although it is well known that ATM and ATR are required to maintain genomic integrity, the precise roles of ATM and ATR during brain development are not fully understood, especially related to neuropathology such as ataxia, neurodegeneration, and microcephaly observed in human patients.

Telomere dysfunction is one of the endogenous sources to induce DNA damage response (DDR), since the unprotected telomere ends could be recognized as DNA strand breaks (de Lange 2005). To protect this DNA region, the telomere ends are coated with the Shelterin complex, which is composed of several proteins including telomeric repeat binding factor 2 (TERF2) and protection of telomeres 1 (POT1), to prevent telomere attrition and inappropriate DDR induction (de Lange 2005; Palm and de Lange 2008). TERF2 homodimers bind to double-stranded regions of telomeres and inactivation of TERF2 triggers ATM-dependent DDR signals, whereas POT1 protects the 3′ single-stranded overhang and its inactivation initiates ATR-dependent DDR signals at the cellular level (Karlseder et al. 1999; Zhang et al. 2006, 2007; Denchi and de Lange 2007; Sfeir and de Lange 2012). The exposed telomere ends as a result of *Terf2* inactivation were processed by *DNA ligase IV (Lig4)* which is the ligase for the canonical Non-homologous end-joining repair (NHEJ) pathway for DSBs (Celli and de Lange 2005; Lee et al. 2016; Smogorzewska et al. 2002). The involvement of ATM and ATR in DDR induced by telomere dysfunction was mutually exclusive in this context.

However, selective deletion of the *Pot1a* gene during mouse brain development resulted in *Atm*-dependent neurological phenotypes including cerebellar defects, suggesting that telomere dysfunction might induce DDR in a tissue-specific manner during neurogenesis (Lee et al. 2014). For the current study, we asked whether telomere dysfunction due to *Terf2* inactivation also induces neuro-specific DDR associated with *Atm* or *Atr* signaling pathway during neurogenesis.

## Materials and methods

### Animals

Floxed *Terf2* animals (Karlseder et al. 1999; Celli and de Lange 2005) were purchased from the Jackson Laboratory (JAX #006568). Germline deletion of the *Terf2* gene caused embryonic lethality at mid-gestation in the mouse (Celli and de Lange 2005). In order to restrict inactivation of the *Terf2* gene in the nervous system during development, *Terf2* ^*LoxP/*+^ animals were interbred with *Nestin*-*Cre* animals (JAX #003771) or *Emx1*-*Cre* animals (JAX #005628). *Cre* recombinase expression driven by the *Nestin* promoter is active around embryonic day (E) 11 throughout the neural progenitors, whereas the *Emx1* promoter is activated only in the dorsal telencephalon and the hippocampal progenitors around E10 during mouse embryogenesis (Gorski et al. 2002; Graus-Porta et al. 2001; Tronche et al. 1999). *Terf2* ^*LoxP/LoxP*^ *;Nestin*-*Cre* and *Terf2* ^*LoxP/LoxP*^ *;Emx1*-*Cre* mice were obtained through a proper breeding scheme. Conditional knockout animals could not be used for breeding. *Terf2* ^*LoxP/*+^ *;Nestin*-*Cre* or *Emx1*-*Cre* animals did not show any discernable defects or shortened life span, and they were fertile. So these animals were included in control groups. Mutant alleles of *Atm*, *Atr* ^*LoxP*^, *Trp53* ^*LoxP*^, *Lig4* ^*LoxP*^, and *Pot1a* ^*LoxP*^ genes and the polymerase chain reaction (PCR) conditions for genotyping were as previously described (Lee et al. 2001, 2012b, 2014; Shull et al. 2009). All animals were maintained in a mixed strain of C57BL/6 X 129 genetic background. Since we could not find any gender difference of DDR in the brain before (Lee et al. 2001, 2012a, 2014), we analyzed the experimental materials regardless of gender for the current study. Also *Nestin*-*Cre* expression was maintained in females for breeding to minimize any ectopic Cre recombinase activity outside of the nervous system. Genotypes of genetically engineered animals were determined by a routine PCR method using the following primers: *Terf2* (Forward: 5′ ccaaccagggatacacagtga, Reverse: 5′ atccgtagttcctcttgtgtctg), *Pot1a* (Forward: 5′ ctcgaattccatctcctcccagtactctctcag, Reverse: 5 ggaactggtacgtatcagtgtgtgtgg), *Atm* wildtype ( WT) allele (Forward: 5′ gcctgtatcttctatgtgcaccgtcttcgc, Reverse: 5′ ggtgcggtgtggatgggactggagg), *Atm* targeted allele (Forward: 5′ gtgatgacctgagacaagatgctgtc, Reverse: 5′ gggaagacaatagcaggcatgc), *Atr* (Forward: 5 tacattttagtcatagttgcataacac, Reverse: 5 cttctaatcttcctccagaattgtaaaagg), *Lig4* (Forward: 5′ atcgctcttgtcccagtacacctgc, Reverse: 5 gtgcattaaatggagtgctgtgc), *Trp53* (Forward: 5′ cacaaaaacaggttaaacccag, Reverse: 5′ agcacataggaggcagagac).

PCR products for *Terf2* genotyping were amplified for 35 cycles of 94 °C for 30 s, 60 °C for 45 s, and 72 °C for 45 s. PCR products for WT and floxed alleles (*LoxP*) of the *Terf2* gene were 233 and 300 bp, respectively.

All of animal materials for experiments were courteously provided by Dr. Peter McKinnon (St. Jude Children’s Research Hospital, USA). The presence of a vaginal plug was indicated as E.5 and the day of birth as postnatal day (P) 0. All animals were housed in an AAALAC accredited facility and were maintained in accordance with the National Institutes of Health ‘Guide for the Care and Use of Laboratory Animals’. All procedures for animal use were approved by the Institutional Animal Care and Use Committee.

### Histology

Histopathological procedures were performed as previously described (Lee et al. 2012a, 2014). In brief, both embryos and brains were collected at indicated time points after the fixation step using 4% phosphate-buffered paraformaldehyde. Cryosections of embryos or brains at 10 μm were collected using an HM500M (Microm) or an MEV (SLEE medical GmbH) cryostat for pathological analysis. Hematoxylin and Eosin (H/E), and Nissl staining were carried out in a routine procedure. Immunoreactivity was visualized by either colorimetric detection using the VIP substrate kit (Vector Labs) reactive with biotinylated secondary antibodies or fluorometric detection using FITC/CY3 conjugated secondary antibodies (Jackson Immunologicals) after incubation with primary antibodies. Counterstaining was done with 0.1% methyl green in 0.1 M sodium acetate buffer for colorimetric staining followed by mounting with DPX (Sigma) or DAPI/PI counterstaining mounting medium (Vector Laboratories) for fluorometric staining.

Antibodies used for this study were Calbindin (mouse, 1:2000, Sigma-Aldrich), Ctip2 (rat, 1:100, Abcam), Cux1 (rabbit, 1:100, Santa Cruz), Foxp2 (rabbit, 1:500, Abcam), GFAP (mouse, 1:500, Sigma-Aldrich), γ-H2AX (rabbit, 1:100, Cell signaling), γ-tubulin (mouse, 1:1000, Sigma-Aldrich), Histone H3phosphoS10 (rabbit, 1:2000, Cell signaling), MBP (myelin basic protein, rabbit, 1:200, Abcam), Myelin-PLP (rabbit, 1:200, Abcam), NeuN (mouse, 1:500, Millipore), Neuronal Class III β tubulin (clone TUJ1, mouse, 1:1000, Covance Research), Tbr1 (rabbit, 1:500, Abcam). Depending on primary antibodies, the citric acid-based antigen retrieval method was applied to enhance immunoreactive signals.

TUNEL (terminal deoxynucleotidyl transferase dUTP nick end labeling) assay using Apoptag (Chemicon) was applied to measure apoptosis. Multiple histological slides were examined and imaged using an Axio Imager A1 microscope (Zeiss) or B600TiFL (Optika). Microscopic images were captured and processed using Photoshop (v.CS6.5, Adobe).

### Telomere in situ using telomere-PNA probe

To detect telomere in situ, tissue sections were dehydrated in alcohol series, denatured at 80 °C for 4 min, followed by incubation for 4 h in the dark at room temperature with CY3-CCCTAACCCTAACCCTAA peptide nucleic acid (PNA) telomere probe (0.7 μg/ml, Panagene) in 70% formamide, 10 mM Tris–HCl pH 7.5, 5% MgCl_2_ buffer (82 mM Na_2_HPO_4_, 9 mM citric acid, 25 mM MgCl_2_), and 1% blocking reagent (Roche). Then, sections were washed with Solution I (70% formamide, 10 mM Tris–HCl pH 7.2, 0.1% BSA) and Solution II (0.05 M Tris–HCl pH 7.2, 0.15 M NaCl, 0.05% Tween 20) several times. After washing, slides were mounted with DAPI-containing aqueous mounting medium (Vector). Images were captured and analyzed as described in the “Histology” method section.

## Results

### *Terf2* was essential for brain development

It has been reported that germline deletion of the *Terf2* gene caused embryonic lethality at mid-gestation in the mouse (Celli and de Lange 2005). So, we restricted *Terf2* inactivation to the nervous system by cross-breeding of the *Terf2* floxed animal model with either *Nestin*-*Cre* (*Terf2* ^*LoxP/LoxP*^ *;Nestin*-*Cre*, hereafter *Terf2* ^*Nes*-*Cre*^) or *Emx1*-*Cre* (*Terf2* ^*LoxP/LoxP*^ *;Emx1*-*Cre*, hereafter *Terf2* ^*Emx1*-*Cre*^) animal lines during development.

**Fig. 1:**
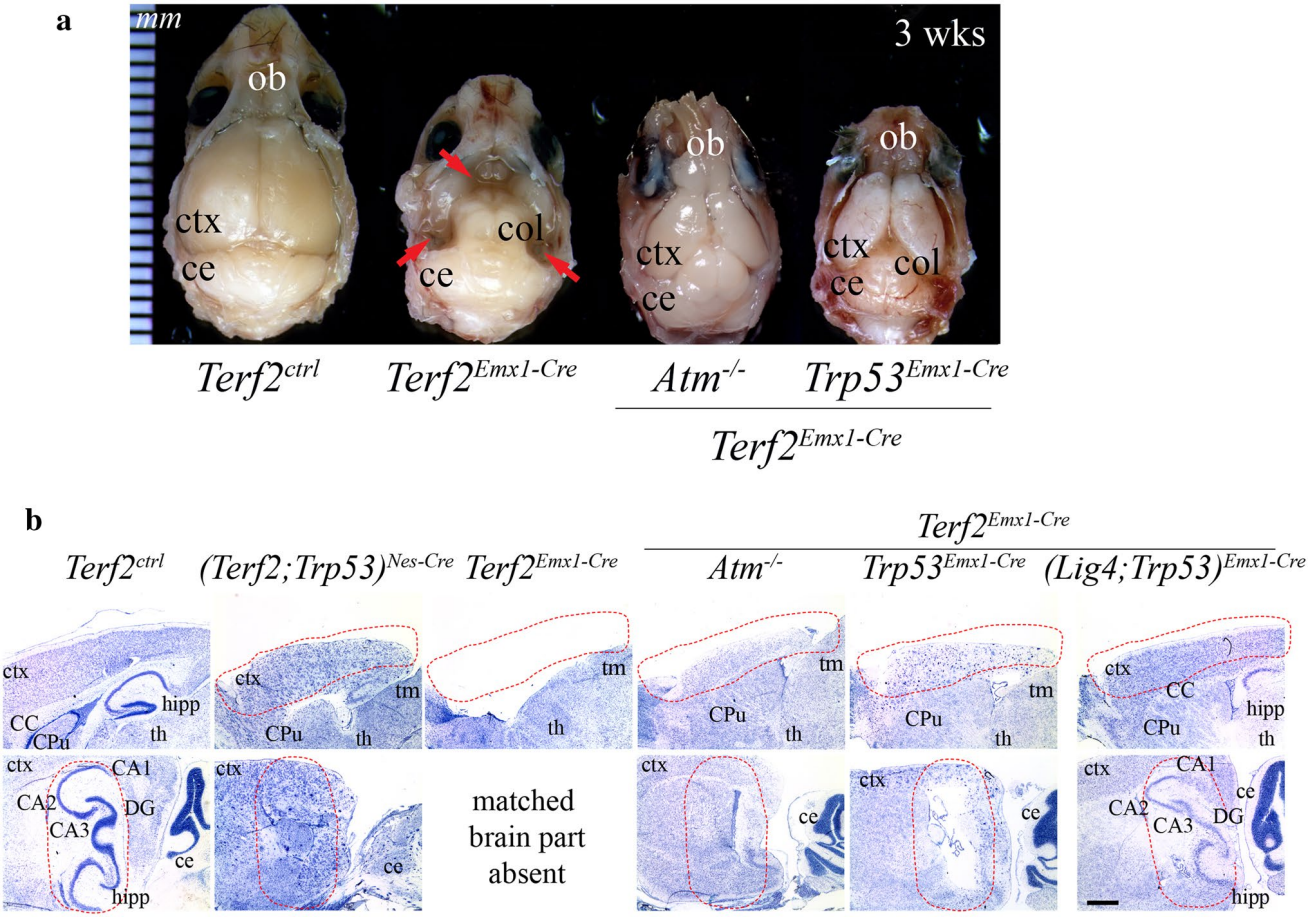
*Terf2* inactivation during neurogenesis resulted in obliteration of the brain structure. **a** The view from the top of *Terf2* conditional knockout brains at 3 weeks of age. The skull was partially removed to show the brain structure. The eyeballs were intact. The control brain was fully developed, yet the *Terf2* ^*Emx1*-*Cre*^ brain had the cerebral cortical part missing in which the *Emx1* promoter-driven *Cre* recombinase was expressed, so the colliculus (col) was fully exposed. *Red arrows* indicate the part lost in the *Terf2* ^*Emx1*-*Cre*^ brain. These missing brain parts were partially restored in both *Atm* and *Trp53*-null backgrounds. *ce* cerebellum, *col* colliculus, *ctx* cerebral cortex, *ob* olfactory bulb. *Ctrl* control, the *ruler* in mm. **b** Nissl staining of the mature brains. The sagittal (*upper panel*) and parasagittal (*lower panel*) views of 3-week-old brains in different genetic backgrounds. *Upper panel* the brain areas expressing *Cre* recombinase controlled by the *Emx1* promotor were completely missing (*Terf2* ^*Emx1*-*Cre*^), but the partial recovery of the brain areas was observed in *Atm-*, *Trp53-* and *Lig4/Trp53*-null backgrounds (*Terf2* ^*Emx1*-*Cre*^ *;Atm* ^−*/*−^, *(Terf2;Trp53)* ^*Emx1*-*Cre*^ and *(Terf2;Lig4;Trp53)* ^*Emx1*-*Cre*^). Also *Trp53* deficiency rescued embryonic lethality as well as partially the missing brain structure in *Terf2* ^*Nes*-*Cre*^ animals. *Lower panel* the hippocampus, one of the important brain structures for memory, in the conditional knockout brains was not formed. Either *Atm* or *Trp53* deficiency was not sufficient enough to restore the hippocampal structure. However, the *(Terf2;Lig4;Trp53)* ^*Emx1*-*Cre*^ brain displayed a partial recovery of the hippocampus. In the parasagittal section, the matched brain part containing the hippocampus in the *Emx*-1 *Cre* background was completely gone, since the *Emx1*-*Cre* was expressed in this particular brain structure during neurogenesis. The *dotted red lines* demarcate some brain areas affected by *Terf2* inactivation, such as the cerebral cortex and hippocampus. *CC* corpus callosum, *ce* cerebellum, *Cpu* caudate putamen, *ctx* cerebral cortex, *th* thalamus, *hipp* hippocampus (*CA1/2/3* Cornu Ammonis areas 1/2/3, *DG* dentate gyrus), *tm* tectum. The *scale bar* is 1.2 mm

Although the *Terf2* gene was targeted only in the nervous system during mouse development, all of *Terf2* ^*Nes*-*Cre*^ mice were born dead without a proper brain structure (data not shown). The overall body size of *Terf2* ^*Nes*-*Cre*^ animals was comparable to that of controls at birth (data not shown). On the other hand, *Terf2* ^*Emx1*-*Cre*^ animals were born at normal Mendelian ratio. These mutant animals became runted and succumbed around 1 month after birth. The *Terf2* ^*Emx1*-*Cre*^ brains showed complete loss of the brain region in which *Emx1*-*Cre* is expressed (Fig. 1a, b). *Terf2* ^*LoxP/*+^ *;Nestin*-*Cre* or *Emx1*-*Cre* animals did not show any discernable neurological defects, so these genotypes of animals alone with WT animals were considered as control groups.

### *Terf2* inactivation induced massive neural apoptosis during development

**Fig. 2:**
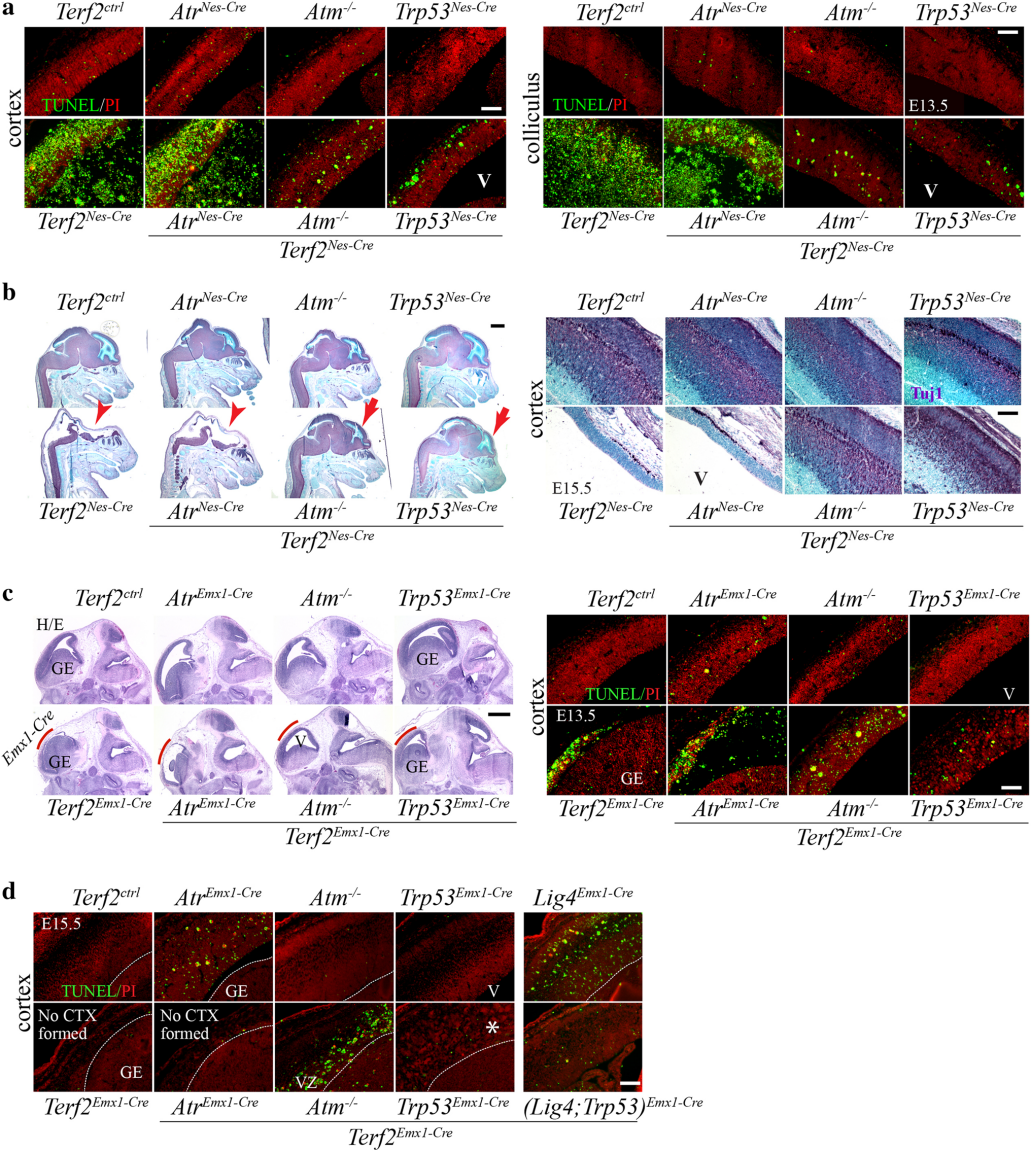
Massive apoptosis occurred in the *Terf2*-deficient developing brain. **a** TUNEL analysis to detect neural apoptosis. The embryonic forebrain (cortex) and colliculus area at embryonic day (E) 13.5 with TUNEL staining. The controls (*Terf2* ^*ctrl*^ *, Atr* ^*Nes*-*Cre*^, *Atm* ^−*/*−^ and *Trp53* ^*Nes*-*Cre*^) showed only the basal levels of TUNEL positivity (green staining), and there was no difference among the control groups. However, massive apoptosis was observed in the *Terf2* ^*Nes*-*Cre*^ and *(Terf2;Atr)* ^*Nes*-*Cre*^ developing brains, while a dramatic reduction of programmed cell death was evident in both *Terf2* ^*Nes*-*Cre*^ *;Atm* ^−*/*−^ and *(Terf2;Trp53)* ^*Nes*-*Cre*^ embryonic cortex and colliculus. Green fluorescent apoptotic debris were falling off inside of the ventricle (*V*) in the *Terf2* ^*Nes*-*Cre*^ and *(Terf2;Atr)* ^*Nes*-*Cre*^ embryonic brains. *Ctrl* control. **b** Tuj1 (Neuron-specific class III beta-tubulin, marker for postmitotic neurons) positive immuno-staining. *Left panel* the heads of embryos (at *E15.5*) with Tuj1 immunoreactivity (*purple color*) at low magnification show that the *Terf2* ^*Nes*-*Cre*^ and *(Terf2;Atr)* ^*Nes*-*Cre*^ brains were completely gone by E15.5 (*red arrowheads*), yet the brain structure was partially rescued in both *Atm* and *Trp53* deficiency (*red arrows*). The ventricular zone was not positive for Tuj1 immunoreactivity, so methyl green staining (counterstaining) was visible in this zone. *Right panel* high magnified views of the forebrains (cortex) in experimental groups. The *Terf2* ^*Nes*-*Cre*^ and *(Terf2;Atr)* ^*Nes*-*Cre*^ embryos did not have any brain structure which was positive for Tuj1 immuostaining (*purple color*). Two pictures of *Terf2* ^*Nes*-*Cre*^ and *(Terf2;Atr)* ^*Nes*-*Cre*^ heads show only a part of the skull and scalp. The missing brain parts were restored in both *Atm* and *Trp53* null backgrounds (*Terf2* ^*Nes*-*Cre*^ *;Atm* ^−*/*−^ and *(Terf2;Trp53)* ^*Nes*-*Cre*^). Methyl green was used for counterstaining so that the ventricular zone could be identifiable. *V* ventricle. **c** H/E and TUNEL staining of the *Emx1*-*Cre* expressing brains at E13.5. *Left panel* Hematoxylin/Eosin (H/E) staining of the embryonic brains shows that the *Cre* expressing area (*red line*) was gone in *Terf2* ^*Emx1*-*Cre*^ and *(Terf2;Atr)* ^*Emx1*-*Cre*^ embryos, but the affected brain area was partially restored in both *Atm* ^−*/*−^ and *Trp53* ^*Emx1-Cre*^ backgrounds. The control animals including *Terf2* ^*ctrl*^ *, Atr* ^*Emx1*-*Cre*^ *, Atm* ^−*/*−^ and *Trp53* ^*Emx1*-*Cre*^ brains did not show any morphological defects. The ganglionic eminence (GE) was intact in the (*Terf2)* ^*Emx1*-*Cre*^ embryonic brains, since *Emx1*-*Cre* is not expressed in the GE. The *red lines* demarcate the *Emx1*-*Cre* expressing area. *GE* ganglionic eminence, *V* ventricle. *Right panel* apoptosis detected by TUNEL (*green color*) shows increased apoptosis in the *Emx1*-*Cre* expressing area of the *Terf2* conditional knockout brains (*Terf2* ^*Emx1*-*Cre*^ and *(Terf2;Atr)* ^*Emx1*-*Cre*^), but not in the GE. *Atm* and *Trp53* deficiency (but not *Atr* deficiency) attenuated apoptosis resulting from *Terf2* inactivation in the developing brain. **d** Apoptosis measured by TUNEL at E15.5. *Terf2*-inactivated brain parts were gone completely in the *Terf2* ^*Emx1*-*Cre*^ and *(Terf2;Atr)* ^*Emx1*-*Cre*^ embryos by E15.5 (No CTX formed). Even though the missing part in the developing brain was partially restored, the *Terf2* ^*Emx1*-*Cre*^ *;Atm* ^−*/*−^ forebrain (cortex: CTX) showed apoptosis restricted to the ventricular zone (VZ), and there was no significant sign of programmed cell death observed in the *(Terf2;Trp53)* ^*Emx1*-*Cre*^ and *(Terf2;Lig4;Trp53)* ^*Emx1*-*Cre*^ developing brains. However, the restored brain area in a *Trp53*-null background contained irregular cellularity (white asterisk), which was not observed in the *Terf2* ^*Emx1*-*Cre*^ *;Atm* ^−*/*−^ and *(Terf2;Lig4;Trp53)* ^*Emx1*-*Cre*^ embryonic brains. Since *Atr* or *Lig4* inactivation during brain development also induces neuronal cell death, the level of programmed cell death found in the *Atr* ^*Emx1*-*Cre*^ and *Lig4* ^*Emx1*-*Cre*^ embryonic brains was as expected. *GE* ganglionic eminence, *VZ* ventricular zone. The *scale bars* in **b** and **c** *left panels* are 1.2 mm, and the *scale bars* in the rest of the photomicrographs are 200 μm

Loss of the brain regions in which *Cre* recombinase was expressed at birth led us to examine the embryonic brains during neurogenesis. The *Terf2* ^*Nes*-*Cre*^ embryos at E13.5 showed massive apoptosis measured by TUNEL throughout the developing brain (Fig. 2a). As a result, there was no proper brain structure found by E15.5 in a *Nestin*-*Cre* background (Fig. 2b). Similarly, *Terf2* ^*Emx1*-*Cre*^ embryos had only the vestige of the forebrain with residual apoptosis at E13.5 (Fig. 2c), and a complete disappearance of the *Emx1*-*Cre* expressing areas by E15.5 (Fig. 2d), while the ganglionic eminence in which *Emx1*-*Cre* is not expressed was intact during neurogenesis. These data suggest an essential requirement of TERF2 and the importance of telomere stability for cell survival during neurogenesis.

### *Atm* and *Trp53*, but not *Atr*, inactivation rescued neural apoptosis resulting from *Terf2* deficiency

Previously, it has been demonstrated that DNA damage triggered by *Terf2* inactivation in vitro induced *Atm*-dependent *Atr*-independent DDR (Karlseder et al. 1999; Celli and de Lange 2005; Zhang et al. 2006, 2007; Denchi and de Lange 2007). So, next we tested the in vivo involvement of *Atm* and *Atr*, as well as *Trp53*, a common downstream substrate, in lethality and massive neural apoptosis resulting from *Terf2* inactivation during neurogenesis. Floxed *Terf2* animals were cross-bred with *Atr* (conditional inactivation: *Atr* ^*Nes*-*Cre*^ and *Atr* ^*Emx1*-*Cre*^), *Atm* (germline inactivation: *Atm* ^−*/*−^) or *Trp53* (conditional inactivation; *Trp53* ^*Nes*-*Cre*^ and *Trp53* ^*Emx1*-*Cre*^) animals. Similar to the in vitro situation (Denchi and de Lange 2007; Karlseder et al. 1999), *Atr* inactivation did not have any influence on the phenotypes observed in both *Terf2* ^*Nes*-*Cre*^ and *Terf2* ^*Emx1*-*Cre*^ animals (Fig. 2). *Terf2* ^*Emx1*-*Cre*^ *;Atm* ^−*/*−^ and *(Terf2;Trp53)* ^*Emx1*-*Cre*^ animals also did not show any discernible improvement in gross phenotypes including smaller body size and shorter life span observed in the *Terf2* ^*Emx1*-*Cre*^ animals (data not shown).

**Fig. 3:**
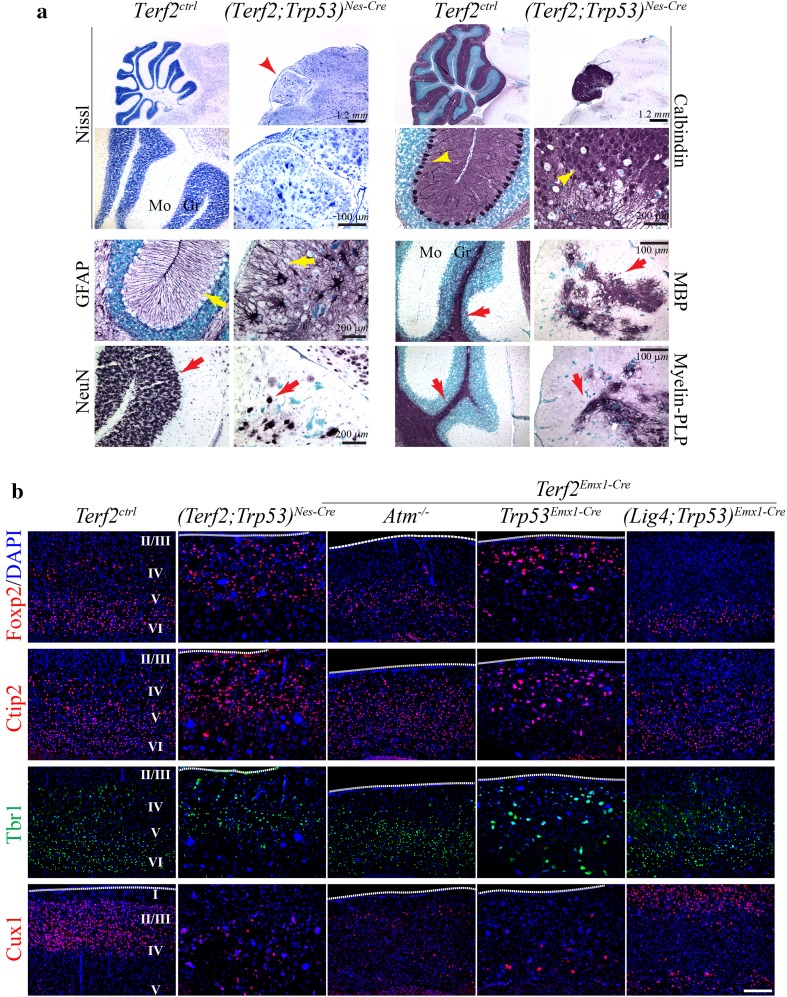
*Terf2* inactivation disrupted the brain structure. **a** Developmental defects in the *(Terf2;Trp53)* ^*Nes*-*Cre*^ cerebellum. Nissl staining indicates that the *(Terf2;Trp53)* ^*Nes*-*Cre*^ cerebellum was much smaller (*red arrowhead*) and the size of cells inside was bigger and not organized. The Purkinje cell layer (*yellow arrowhead*) detected by Calbindin immunostaining displayed the complete misalignment of the layer and could not be found as a single cell layer as shown in the control brain. Glial fibrillary acidic protein (GFAP) immunoreactivity shows the disruption of the Bergmann glial network (*yellow arrow*). The populations of neurons (NeuN immuno-positive) and oligodendrocytes (MBP and Myelin-PLP immuno-positive) were strikingly reduced, and the structure was disorganized (*red arrows*). These cerebellar defects lead to severe ataxia in the *(Terf2;Trp53)* ^*Nes*-*Cre*^ animals (see the accompanied video clip). *Mo* the molecular layer in the cerebellum, *Gr* the granule cell layer in the cerebellum, *Ctrl* control. *Scale bars* are as indicated in the figures. **b** Disruption of the six-layered cortical structure. The layered cortical structure in the 3-week-old brain was examined with four different markers; the upper layers—Cux1; the lower layers—Tbr1, Ctip2, Foxp2. The rescued part of the *Terf2-*null cortex in either *Atm* or *Trp53* deficiency (*(Terf2;Trp53)* ^*Nes*-*Cre*^ *, Terf2* ^*Emx1*-*Cre*^ *;Atm* ^−*/*−^ and *(Terf2;Trp53)* ^*Emx1*-*Cre*^) did not form the upper layers (Cux1 immuno-positive), and the layers IV, V, VI were located to the upper part of the cortex. In contrast, the *(Terf2;Lig4;Trp53)* ^*Emx1*-*Cre*^ cortex displayed all six layers including the upper layers (Cux1 immuno-positive). The size of immunopositive cells to the layer markers in the *Terf2/Trp53* dual null cortex was much bigger and less organized than that of controls. *Roman numeric signs* indicate the cortical layers. The *white dotted lines* indicate the surface of the cerebral cortex. The *scale bar* is 100 μm

In a *Nestin*-*Cre* background, *Atm* inactivation could not rescue prenatal lethality resulting from *Terf2* inactivation, while *(Terf2;Trp53)* ^*Nes*-*Cre*^ animals were born alive. Noticeably, *(Terf2;Trp53)* ^*Nes*-*Cre*^ animals displayed severe ataxia, hence could live only up to the time of weaning (supplementary video clip). This severe ataxic phenotype resulted from the malformation of the cerebellum which was very small and did not have any organized structure of the cerebellum, particularly a complete disruption of the Purkinje cell layer (Fig. 3a). Yet the mutant cerebellum contained all of the cellular components including Purkinje cells (Calbindin immuno-positive), Bergmann glia (glial fibrillary acidic protein (GFAP) immuno-positive), and oligodendrocytes (myelin basic protein (MBP) and myelin proteolipid protein (Myelin-PLP) immuno-positive), except granule cells (NeuN immuno-positive) (Fig. 3a). Tumorigenicity in the nervous system of the (*Terf2;Trp53)* ^*Nes*-*Cre*^ animals could not be evaluated because of a short life span.

Although both *Atm* and *Trp53* inactivation could reduce apoptosis dramatically in the *Terf2*-null brains (Fig. 2), there was a difference between these two genetic backgrounds. Neural cell death in both *Terf2* ^*Nes*-*Cre*^ and *Terf2* ^*Emx1*-*Cre*^ embryos had disappeared by E15.5 in a *Trp53*-null background, whereas *Atm* inactivation could not stall most of the apoptosis in the ventricular zone (VZ) where neural progenitor cells are located in (Fig. 2d).

### The multiple defects were found in the *Terf2*-null brains

Although neuron-specific class III β-tubulin (Tuj1), which is a marker for postmitotic neurons, showed a normal distribution in the developing brains of the *Terf2/Atm* and *Terf2/p53* double null embryos (Fig. 2b), the restored portion in the mature brains exhibited several faulty features. *Trp53* inactivation led to better restoration of the missing parts in the *Terf2*-null brain than *Atm* deficiency did. However, the restoration was still incomplete (Fig. 1b). The hippocampus was not restored in the *Terf2/Atm* and *Terf2/Trp53* double null brains (Fig. 1b).

Next, we analyzed the six-layered cerebral cortical structure created in an inside-out manner during development (Greig et al. 2013). The Foxp2, Ctip2, and Tbr1 immuno-positive neurons, which are generated during an early stage of cortical development and localized in the lower layers of the cerebral cortex in normal development, were found in the upper cortical part of the *(Terf2;Trp53)* ^*Nes*-*Cre*^ and *(Terf2;Trp53)* ^*Emx1*-*Cre*^ brains (Fig. 3b). Similarly, the *Terf2* ^*Emx1*-*Cre*^ *;Atm* ^−*/*−^ cortex showed mis-localization of Ctip2 and Tbr1 immuno-positive neurons. Furthermore, there were no proper upper layers formed (layers I, II, and III), which were Cux1 immuno-positive, in both the *Terf2/Atm* and *Terf2/Trp53* double null cortices, suggesting that the *Terf2-*null developing brain could not generate cortical neurons at the later stage during development in both *Atm-* and *Trp53*-deficient backgrounds (Fig. 3b).

**Fig. 4:**
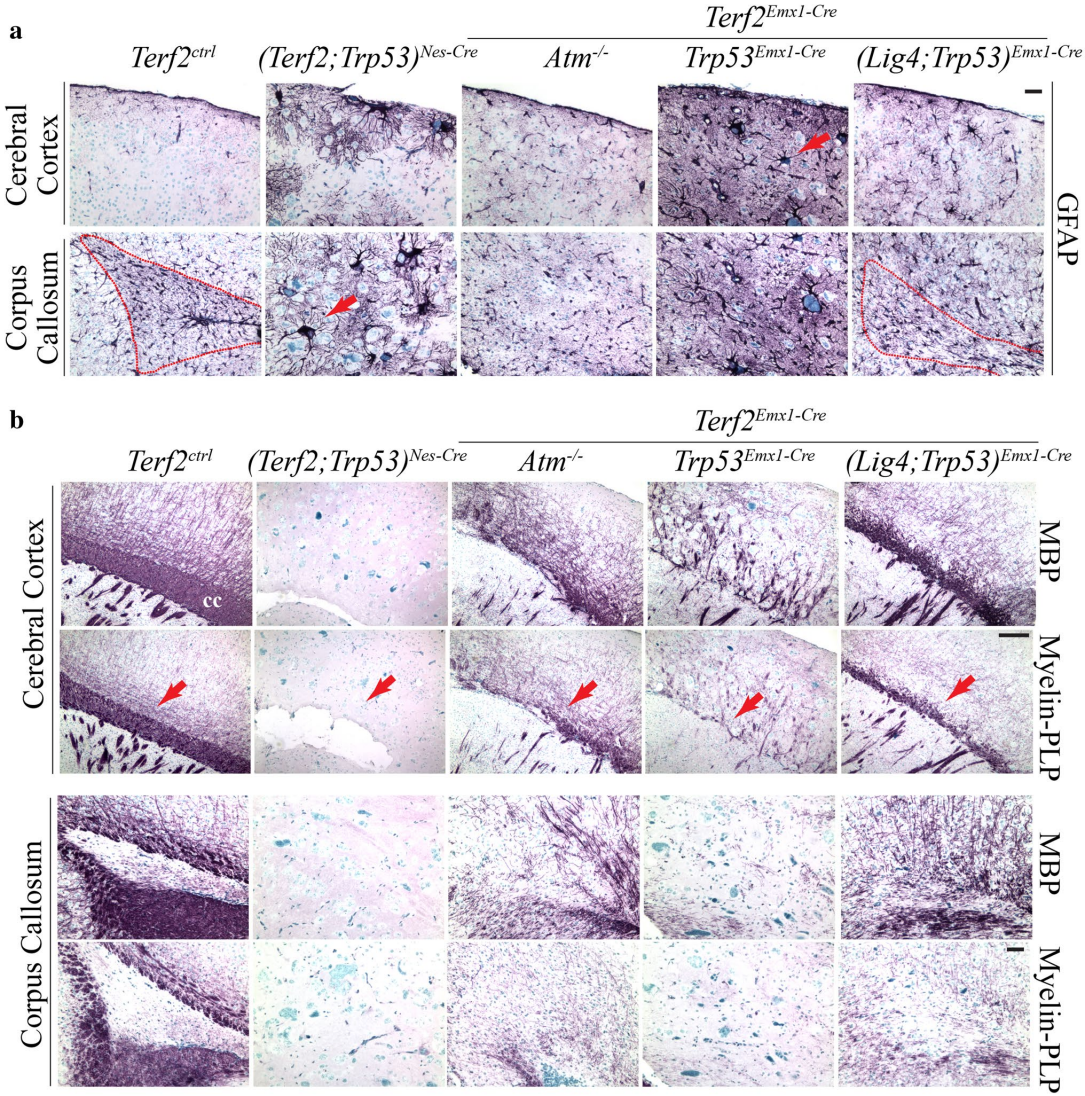
Defective glia network was formed in the mutant brain. **a** Increased immunoreactivity of GFAP in the *Terf2* mutant brain. The *Terf2*-null brains in both *Atm-* and *Trp53-*deficient backgrounds showed the high level of GFAP immuno-staining compared to that of the control brain, which is a common feature found in the brain of genomic instability animal models. The corpus callosum, which is a glia-rich structure, was not defined in the *Terf2*-null brain (*(Terf2;Trp53)* ^*Nes*-*Cre*^ *, Terf2* ^*Emx1*-*Cre*^ *;Atm* ^−*/*−^ and *(Terf2;Trp53)* ^*Emx1*-*Cre*^). The *(Terf2;Lig4;Trp53)* ^*Emx1*-*Cre*^ brain showed partial recovery of the corpus callosum. The *red dotted lines* demarcate the normal corpus callosum in the control brain and the partially restored corpus callosum in the *(Terf2;Lig4;Trp53)* ^*Emx1*-*Cre*^ brain. The size of GFAP immuno-positive cells in the *Terf2/Trp53* double null brains, not in both *Terf2/Atm* double and *Terf2/Lig4/Trp53* triple deficient brains, was bigger than that of controls (*red arrows*). The *scale bar* is 200 μm. **b** Defective oligodendrocyte network in the *Terf2* mutant brain. Oligodendrocytes were detected by two different markers: MBP (myelin basic protein) and Myelin-PLP (proteolipid protein). The *Terf2* ^*ctrl*^ cerebral cortex displayed a rich network of oligodendrocytes, particularly in the corpus callosum. However, the *Terf2* conditional knockout brains in all different genetic backgrounds showed a dramatic reduction of immunopositivity to MBP/Myelin-PLP, and agenesis of the corpus callosum (*red arrows*), in contrast to the increased astrocyte population in the *Terf2*-null brains (A *panel above*). And the genetic combination with *Atm* or *Trp53* deficiency could not improve this defect. However, *Lig4* inactivation resulted in partial recovery of the corpus callosum in the *(Terf2;Trp53)* ^*Emx1*-*Cr*e^ brain. The corpus callosum shown in the *lower panel* was the posterior part of the corpus callosum. The *scale bars* are 100 μm

Increased GFAP immunoreactivity was one of common features found in the brains of genomic instability animal models (Shull et al. 2009; Lee et al. 2012a, b). So we tested whether *Terf2*-deficient brains also exhibit increased GFAP immunoreactivity as a sign of genomic instability. Indeed the *Terf2-*null cortex showed increased GFAP immunopositivity in both *Atm-* and *p53*-null backgrounds (Fig. 4a). Interestingly, the size of GFAP immuno-positive cells in *Terf2/Trp53* double null brains was relatively big (Fig. 4a). On the contrary, the dramatic reduction of oligodendrocyte population which was visualized by MBP and Myelin-PLP immunoreactivity was found in the *Terf2/Atm* and *Terf2/Trp53* double null cortices (Fig. 4b). However, all of the *Terf2-*null cortices were in the absence of the corpus callosum (CC) structure, which functions as a communication path between two cerebral hemispheres (Fig. 4a, b).

### *Trp53* inactivation resulted in multinucleated giant neural cells in the *Terf2*-null brain

**Fig. 5:**
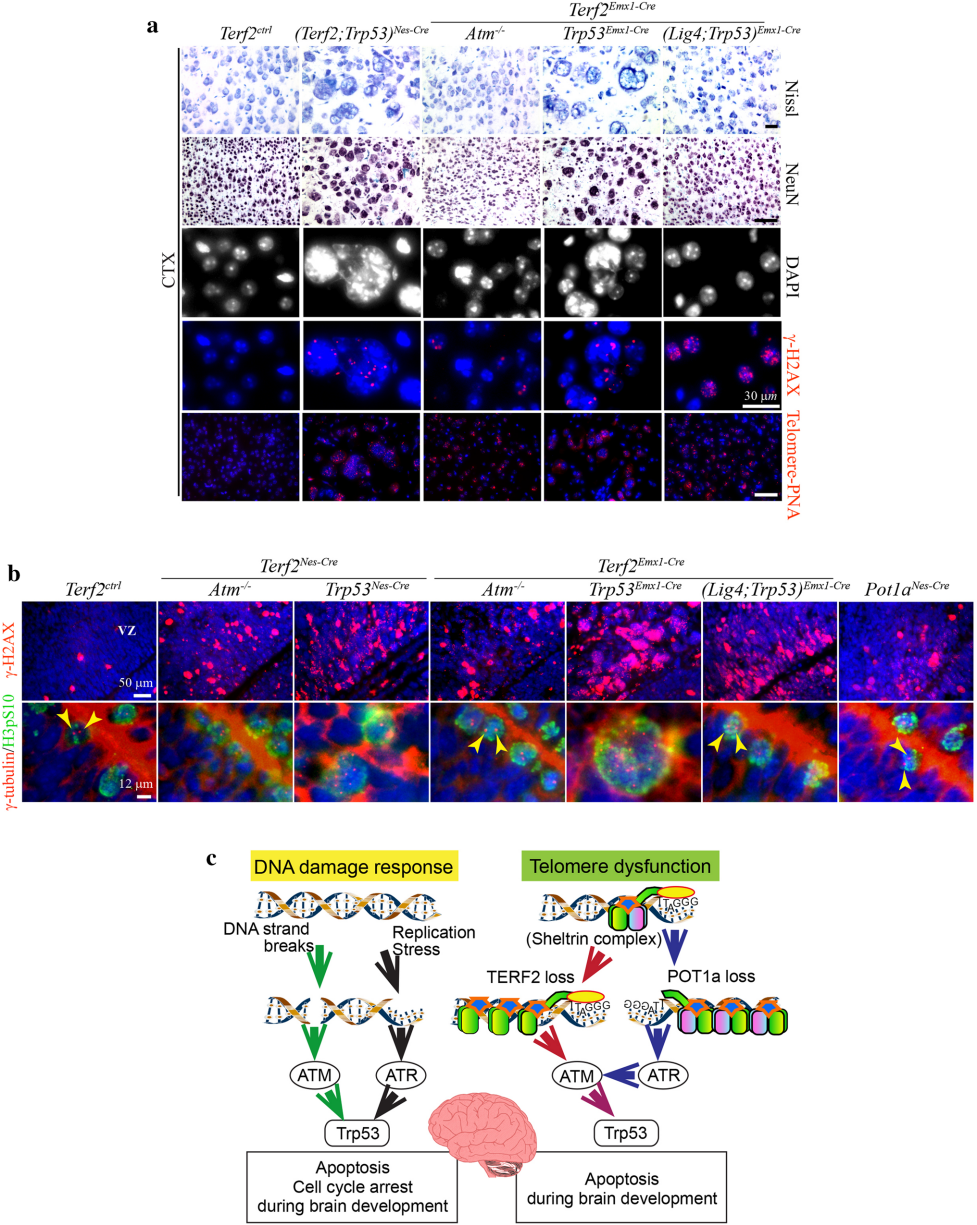
The multinucleated giant neural cells were found only in the *Terf2*/*Trp53* double null brain. **a** Giant neural cells in the *Terf2/Trp53* double null brains and a sign of DNA damage in the *Terf2* null brain. The neural cells including neurons in the brain, particularly the cerebral cortex (CTX), were examined by Nissl staining and NeuN immunoreactivity. Giant neural cells were found only in the *(Terf2;Trp53)* ^*Nes*-*Cre*^ and *(Terf2;Trp53)* ^*Emx1*-*Cre*^ cortices. Giant neural cells had multiple nuclei in a single cell visualized by DAPI staining in the *Terf2/Trp53* double deficient cortex. This kind of giant neural cells were not observed in the *Terf2* ^*Emx1*-*Cre*^ *;Atm* ^−*/*−^ and *(Terf2;Lig4;Trp53)* ^*Emx1*-*Cre*^ cortices. DNA damage or breaks could be visualized by immunostaining of phosphorylated H2AX (γ-H2AX) as foci formation in the nucleus. γ-H2AX foci were abundant in the giant nuclei of the *Terf2/Trp53* double null cortices. Since *Lig4* deficiency itself results in accumulation of γ-H2AX foci, the *(Terf2;Lig4;Trp53)* ^*Emx1*-*Cre*^ cortex showed strong foci formation in the nucleus. Telomeres were visualized by hybridization with Telomere-PNA probe conjugated with CY3 (*red*). Only the *Terf2*-null cortices in all different genetic backgrounds showed the high level of PNA probe positivity indicating that the PNA-telomere probe was hybridized better with telomeres in the *Terf2*-null brains. The blue staining was DAPI staining for γ-H2AX immunostaining and PNA-telomere in situ detection. The *scale bars* are 100 μm except γ-H2AX immunostaining. **b** The high level of γ-H2AX foci and big chromosomes in the *Terf2*-null embryonic cortex during embryogenesis. DNA damage visualized by γ-H2AX foci was evident in the *Terf2*-null embryonic fore brains in different genetic backgrounds as well as in the *Pot1a* ^*Nes*-*Cre*^ embryonic brain, particularly in the ventricular zone (VZ) of the cortex. A big conglomeration of chromosomes detected by phosphorylated H3 (H3pS10) immunoreactivity (green staining) was observed only in the *Terf2/Trp53* double null metaphase cells (*(Terf2;Trp53)* ^*Nes*-*Cre*^ and *(Terf2;Trp53)* ^*Emx1*-*Cre*^), not in *Terf2/Atm* double and *Terf2/Lig4/Trp53* triple null metaphase cells. Centrosomes were visualized by γ-tubulin immunoreactivity (*yellow arrowheads*). Only *Terf2/Trp53* double null metaphase cells in both *Nestin* and *Emx*-*1 Cre* backgrounds had multiple centrosomes (red staining) in a single-metaphase cell. *Terf2* ^*Nes*-*Cre*^ *;Atm* ^−*/*−^ *, Terf2* ^*Emx1*-*Cre*^ *;Atm* ^−*/*−^ *and (Terf2;Lig4;Trp53)* ^*Emx1*-*Cre*^ neural progenitor cells at metaphase had two centrosomes the same as the control embryonic brain (*yellow arrowheads*). *Pot1a* deficiency (*Pot1a* ^*Nes*-*Cre*^) did not show any similar defects such as a big aggregation of chromosomes and multiple centrosomes. The *scale bars* are as indicated in the figure. **c** A schematic illustration of signaling pathways induced by either DNA damage or telomere dysfunction to trigger apoptosis or cell cycle arrest during brain development. Telomere dysfunction resulting from either TERF2 or POT1a inactivation induces neural apoptosis which is ATM-Trp53 dependent during neurogenesis. *Terf2* inactivation directly activates ATM-dependent signaling, while *Pot1a* inactivation induces ATM activation via ATR signaling during neurogenesis

Furthermore, we noticed the existence of giant neural cells only in the *Terf2/Trp53* double null brains, not in the *Terf2/Atm* double null brains (Figs. 3, 4a, 5a). Each giant cell contained irregular shaped multiple nuclei connected to each other by thin strings (Fig. 5a).

Also, the considerable amounts of DNA damage visualized by phosphorylated H2AX (γ-H2AX) foci formation, which is a good marker for DNA strand breaks, were detected in all of the *Terf2*-null brains, but not in the control brain (Fig. 5a). In addition, telomere in situ visualization using a telomere-PNA probe showed its conjugated CY3 signals in the *Terf2*-null brains (Fig. 5a). These telomere-PNA signals were not noticeable in the control brain, suggesting that telomeres in *Terf2*-null neural cells were readily accessible by the telomere-PNA probe since they were not well protected.

Next we examined whether multinucleated giant neural cells were formed during neurogenesis. Certainly, γ-H2AX foci formation in the *Terf2*-null brains, particularly in the VZ of the developing brains, was dramatically increased in both *Atm-* and *Trp53*-deficient backgrounds (Fig. 5b). However, the chromosome mass at metaphase visualized by phosphorylated H3 immunoreactivity (H3pS10) showed a significant increase only in the *Terf2/Trp53* double null neural progenitors (Fig. 5b). This giant chromosomal mass was not observed in an *Atm-*null background. It is possible that this giant chromosomal aggregation might partially result from incomplete cell division, since multiple centrosomes detected by γ-tubulin immunoreactivity in a single cell were observed only in the *Terf2/Trp53* double null neural cells at metaphase (Fig. 5b). Even though DNA damage detected by γ-H2AX foci was evident, neural progenitor cells with giant chromosomal mass were not observed in the *Pot1a* ^*Nes*-*Cre*^ embryonic brains (Fig. 5b); consequently, there were no multinucleated giant neural cells found in the *Pot1a* ^*Nes*-*Cre*^ mature brains (Lee et al. 2014).

### DNA ligase IV plays a role in neural abnormalities due to *Terf2* deficiency during neurogenesis

Previously it was reported that *Lig4* plays a role in abnormal fusions of telomere ends in *Terf2*-deficient cells (Celli and de Lange 2005; Smogorzewska et al. 2002). So we tested whether *Lig4* inactivation during neurogenesis could alleviate neural abnormalities observed in the (*Terf2/Trp53)* ^*Emx1*-*Cre*^ brain. As described before (Lee et al. 2012a), the *(Lig4;Trp53)* ^*Emx1*-*Cre*^ brain did not show any neural phenotypes related to telomere dysfunction. The triple conditional knockout animals (hereafter *(Terf2;Lig4;Trp53)* ^*Emx1*-*Cre*^) were born alive, but the *(Terf2;Lig4;Trp53)* ^*Emx1*-*Cre*^ animals died around 1 month of age similar to the *(Terf2;Trp53)* ^*Emx1*-*Cre*^ animals.

Neuropathological analysis revealed that the neurological abnormalities were moderately corrected in the *(Terf2;Lig4;Trp53)* ^*Emx1*-*Cre*^ brain. During embryogenesis, the reduction of neural cell death in the *(Terf2;Lig4;Trp53)* ^*Emx1*-*Cre*^ embryos was the same as the *(Terf2;Trp53)* ^*Emx1*-*Cre*^ embryos, most likely due to the effect of *Trp53* inactivation (Fig. 2d). However, proliferating cells at metaphase in the *(Terf2;Lig4;Trp53)* ^*Emx1*-*Cre*^ VZ were similar to those with two centrosomes found in control embryos (Fig. 5b). Consequently, the most obvious correction was disappearance of multinucleated giant cells in the triple conditional null mature brain (Figs. 4a, 5a). In addition, the upper layers of the cerebral cortex, which are Cux1 immuno-positive, were formed in the triple null brain (Fig. 3b). However, this recovery by *Lig4* inactivation was not complete, such as partial reconstruction of the hippocampus and CC compared to the control groups (Figs. 1b, 4).

## Discussion

### Telomere homeostasis is essential for brain development in the mouse

The role of TERF2 and POT1 as components of the Shelterin complex is to protect the telomere ends so that genomic integrity is maintained (Celli and de Lange 2005; Denchi and de Lange 2007; Karlseder et al. 1999). Previously the *Terf2* gene of the mouse was conditionally targeted only in the liver and skin (Bojovic et al. 2013; Lazzerini Denchi et al. 2006; Martinez et al. 2014). *Terf2* inactivation in the basal layer of epidermis using a *K14*-*Cre* animal line did not cause embryonic lethality (Bojovic et al. 2013), while *Terf2* inactivation in epidermal stem cells using a *K5*-*Cre* animal line led to partial embryonic lethality and impaired skin development (Martinez et al. 2014). Similarly, here we demonstrated that a selective inactivation of the *Terf2* gene in the neural progenitor cells resulted in embryonic fatality and loss of brain structures, providing another example that the protective role of TERF2 is important to maintain cellular and organismal viability. This result is clearly different from the animal model for selective inactivation of the *Terf2* gene in the liver that showed normal liver function and regeneration through endoreduplication without cell division (Lazzerini Denchi et al. 2006). The status of cells, such as proliferating vs. resting cells, most likely contributes to this difference in cellular viability as suggested before (Martinez et al. 2014).

Interestingly, there was no similarity of the neural phenotypes between the *Terf2* ^*Nes*-*Cre*^ and *Pot1a* ^*Nes*-*Cre*^ animals (Lee et al. 2014), even though germline deletion of the *Terf2* or *Pot1a* gene in the mouse resulted in embryonic lethality (Celli and de Lange 2005; Hockemeyer et al. 2006; Wu et al. 2006), *Pot1a* inactivation in neural progenitor cells did not induce massive neural apoptosis during embryogenesis, and the neurological defects resulting from *Pot1a* inactivation were restricted mainly to the cerebellum (Lee et al. 2014). Furthermore, *Pot1a* ^*Nes*-*Cre*^ *;Trp53* ^−*/*−^ animals did not show any sign of ataxia. On the contrary, *Terf2* inactivation in the neural progenitor cells induced more global effects throughout the central nervous system. Also it appeared that there was a differentiation defect of late-born neurons originated from *Terf2*-null neural progenitor cells which underwent more cell divisions in a *Trp53*-deficient background.

As illustrated in Fig. 5c, it is possible that the Shelterin complex with all components including POT1 presents only at the end of telomeres where the 3′ single-stranded GC rich overhang is exposed (de Lange 2005; Gramatges and Bertuch 2013; Palm and de Lange 2008). For this reason, the impact resulting from *Terf2* inactivation in the neural progenitor cells was more severe than that of *Pot1a* inactivation in the murine nervous system.

### ATM and Trp53 are key signaling mediators in telomere dysfunction due to *Terf2* inactivation during brain development in the mouse

One of the interesting observations was the entity of multinucleated giant neural cells only in the *Terf2/Trp53* conditional knockout mature brain, possibly resulting from endoreduplication without proper cell division as suggested in other *Terf2* conditional knockout animal models (Lazzerini Denchi et al. 2006; Martinez et al. 2014; Ullah et al. 2009). It had been demonstrated that endoreduplication and mitotic failure in a *Trp53*-dependent manner during telomere crisis lead to polyploid cell accumulation (Davoli and de Lange 2012; Pampalona et al. 2012; Davoli et al. 2010). This defect of multinucleated giant cells was resolved by *Lig4* inactivation, suggesting that the ligation function of LIG4 plays a role in this particular neural defect due to telomere dysfunction resulting from *Terf2* inactivation during brain development.

*Trp53* inactivation suppressed neural apoptosis triggered by *Terf2* inactivation in the entire developing nervous system including the VZ in which big metaphase cells could be formed, whereas *Atm* deficiency could not inhibit programmed cell death in the VZ of the *Terf2*-null embryonic brain. Therefore, multinucleated giant neural cells were not observed in the *Terf2/Atm* knockout brain. Also this result suggests that *Trp53* activation to induce neural apoptosis in the *Terf2*-null VZ was *Atm*-independent. This observation was consistent with the previous reports demonstrating that ATM is involved in neural apoptosis via Trp53 activation in the postmitotic zone, not in the VZ (Lee et al. 2000, 2001; Orii et al. 2006). Apparently, ATR is not the key kinase to activate Trp53 signaling in the *Terf2*-null VZ, since *Atr* inactivation did not have any effect on the neural defects in the *Terf2*-null brain, similar to the in vitro situation (Denchi and de Lange 2007). In addition, there was likely no cross-talk between *Atm* and *Atr* signalings responding to telomere dysfunction resulting from *Terf2* inactivation during brain development, in contrast to the situation of *Pot1a* deficiency in the developing brain (Lee et al. 2014). Alternatively, DNA-dependent protein kinase (DNA-PK) might be involved in the Trp53 signaling in the VZ upon DDR induced by *Terf2* deficiency (Rybanska-Spaeder et al. 2014).

Taken all together in combination with our previous report (Lee et al. 2014), we demonstrated that TERF2 is more critical to maintain telomere homeostasis than POT1a in the developing mouse brain. Telomere dysfunction by *Terf2* inactivation induces the ATM-Trp53 signaling axis to trigger neural apoptosis as a part of the mechanisms to maintain genomic integrity during neurogenesis as illustrated in Fig. 5c. ATM is also activated via ATR signaling by telomere dysfunction due to *Pot1a* inactivation in the same physiological context.

## Electronic supplementary material

Below is the link to the electronic supplementary material.

Supplementary material 1 (MOV 3414 kb)

## Abbreviations

DSBs: DNA double-strand breaks
DDR: DNA damage response
P: Postnatal day
E: Embryonic day
VZ: Ventricular zone
CC: Corpus callosum

## References

[CR1] Bojovic B, Ho HY, Wu J, Crowe DL (2013). Stem cell expansion during carcinogenesis in stem cell-depleted conditional telomeric repeat factor 2 null mutant mice. Oncogene.

[CR2] Celli GB, de Lange T (2005). DNA processing is not required for ATM-mediated telomere damage response after TRF2 deletion. Nat Cell Biol.

[CR3] Davoli T, de Lange T (2012). Telomere-driven tetraploidization occurs in human cells undergoing crisis and promotes transformation of mouse cells. Cancer Cell.

[CR4] Davoli T, Denchi EL, de Lange T (2010). Persistent telomere damage induces bypass of mitosis and tetraploidy. Cell.

[CR5] de Lange T (2005). Shelterin: the protein complex that shapes and safeguards human telomeres. Genes Dev.

[CR6] Denchi EL, de Lange T (2007). Protection of telomeres through independent control of ATM and ATR by TRF2 and POT1. Nature.

[CR7] Gorski JA, Talley T, Qiu M, Puelles L, Rubenstein JL, Jones KR (2002). Cortical excitatory neurons and glia, but not GABAergic neurons, are produced in the Emx1-expressing lineage. J Neurosci.

[CR8] Gramatges MM, Bertuch AA (2013). Short telomeres: from dyskeratosis congenita to sporadic aplastic anemia and malignancy. Transl Res.

[CR9] Graus-Porta D, Blaess S, Senften M, Littlewood-Evans A, Damsky C, Huang Z, Orban P, Klein R, Schittny JC, Muller U (2001). Beta1-class integrins regulate the development of laminae and folia in the cerebral and cerebellar cortex. Neuron.

[CR10] Greig LC, Woodworth MB, Galazo MJ, Padmanabhan H, Macklis JD (2013). Molecular logic of neocortical projection neuron specification, development and diversity. Nat Rev Neurosci.

[CR11] Hockemeyer D, Daniels JP, Takai H, de Lange T (2006). Recent expansion of the telomeric complex in rodents: two distinct POT1 proteins protect mouse telomeres. Cell.

[CR12] Karlseder J, Broccoli D, Dai Y, Hardy S, de Lange T (1999). p53- and ATM-dependent apoptosis induced by telomeres lacking TRF2. Science.

[CR13] Lazzerini Denchi E, Celli G, de Lange T (2006). Hepatocytes with extensive telomere deprotection and fusion remain viable and regenerate liver mass through endoreduplication. Genes Dev.

[CR14] Lee Y, Barnes DE, Lindahl T, McKinnon PJ (2000). Defective neurogenesis resulting from DNA ligase IV deficiency requires Atm. Genes Dev.

[CR15] Lee Y, Chong MJ, McKinnon PJ (2001). Ataxia telangiectasia mutated-dependent apoptosis after genotoxic stress in the developing nervous system is determined by cellular differentiation status. J Neurosci.

[CR16] Lee Y, Katyal S, Downing SM, Zhao J, Russell HR, McKinnon PJ (2012). Neurogenesis requires TopBP1 to prevent catastrophic replicative DNA damage in early progenitors. Nat Neurosci.

[CR17] Lee Y, Shull ER, Frappart PO, Katyal S, Enriquez-Rios V, Zhao J, Russell HR, Brown EJ, McKinnon PJ (2012). ATR maintains select progenitors during nervous system development. EMBO J.

[CR18] Lee Y, Brown EJ, Chang S, McKinnon PJ (2014). Pot1a prevents telomere dysfunction and ATM-dependent neuronal loss. J Neurosci.

[CR19] Lee Y, Choi I, Kim J, Kim K (2016). DNA damage to human genetic disorders with neurodevelopmental defects. J Genet Med.

[CR20] Lovejoy CA, Cortez D (2009). Common mechanisms of PIKK regulation. DNA Repair.

[CR21] Martinez P, Ferrara-Romeo I, Flores JM, Blasco MA (2014). Essential role for the TRF2 telomere protein in adult skin homeostasis. Aging Cell.

[CR22] McKinnon PJ (2012). ATM and the molecular pathogenesis of ataxia telangiectasia. Ann Rev Pathol.

[CR23] McKinnon PJ (2013). Maintaining genome stability in the nervous system. Nat Neurosci.

[CR24] Nam EA, Cortez D (2011). ATR signalling: more than meeting at the fork. Biochem J.

[CR25] Orii KE, Lee Y, Kondo N, McKinnon PJ (2006). Selective utilization of nonhomologous end-joining and homologous recombination DNA repair pathways during nervous system development. Proc Natl Acad Sci USA.

[CR26] Palm W, de Lange T (2008). How shelterin protects mammalian telomeres. Annu Rev Genet.

[CR27] Pampalona J, Frias C, Genesca A, Tusell L (2012). Progressive telomere dysfunction causes cytokinesis failure and leads to the accumulation of polyploid cells. PLoS Genet.

[CR28] Rybanska-Spaeder I, Ghosh R, Franco S (2014). 53BP1 mediates the fusion of mammalian telomeres rendered dysfunctional by DNA-PKcs loss or inhibition. PLoS One.

[CR29] Sfeir A, de Lange T (2012). Removal of shelterin reveals the telomere end-protection problem. Science.

[CR30] Shull ER, Lee Y, Nakane H, Stracker TH, Zhao J, Russell HR, Petrini JH, McKinnon PJ (2009). Differential DNA damage signaling accounts for distinct neural apoptotic responses in ATLD and NBS. Genes Dev.

[CR31] Smogorzewska A, Karlseder J, Holtgreve-Grez H, Jauch A, de Lange T (2002). DNA ligase IV-dependent NHEJ of deprotected mammalian telomeres in G1 and G2. Curr Biol CB.

[CR32] Tronche F, Kellendonk C, Kretz O, Gass P, Anlag K, Orban PC, Bock R, Klein R, Schutz G (1999). Disruption of the glucocorticoid receptor gene in the nervous system results in reduced anxiety. Nat Genet.

[CR33] Ullah Z, Lee CY, Lilly MA, DePamphilis ML (2009). Developmentally programmed endoreduplication in animals. Cell Cycle.

[CR34] Wu L, Multani AS, He H, Cosme-Blanco W, Deng Y, Deng JM, Bachilo O, Pathak S, Tahara H, Bailey SM, Deng Y, Behringer RR, Chang S (2006). Pot1 deficiency initiates DNA damage checkpoint activation and aberrant homologous recombination at telomeres. Cell.

[CR35] Zhang P, Furukawa K, Opresko PL, Xu X, Bohr VA, Mattson MP (2006). TRF2 dysfunction elicits DNA damage responses associated with senescence in proliferating neural cells and differentiation of neurons. J Neurochem.

[CR36] Zhang P, Dilley C, Mattson MP (2007). DNA damage responses in neural cells: focus on the telomere. Neuroscience.

